# Beneficial health effects of Menaquinone‐7 on body composition, glycemic indices, lipid profile, and endocrine markers in polycystic ovary syndrome patients

**DOI:** 10.1002/fsn3.1837

**Published:** 2020-09-04

**Authors:** Firoozeh Tarkesh, Bahia Namavar Jahromi, Najmeh Hejazi, Hamidreza Tabatabaee

**Affiliations:** ^1^ Clinical Nutrition Department School of Nutrition and Food Sciences Shiraz University of Medical Sciences Shiraz Iran; ^2^ Infertility Research Center Shiraz University of Medical Sciences Shiraz Iran; ^3^ Department of OB‐GYN School of Medicine Shiraz University of Medical Sciences Shiraz Iran; ^4^ Nutrition Research Center Department of Clinical Nutrition School of Nutrition and Food Sciences Shiraz University of Medical Sciences Shiraz Iran; ^5^ Department of Epidemiology School of Health Shiraz University of Medical Sciences Shiraz Iran

**Keywords:** body composition, insulin resistance, Menaquinone‐7, polycystic ovary syndrome

## Abstract

**Objective:**

The aim of this study was to investigate the effect of oral vitamin K2 (Menaquinone‐7 [MK‐7]) on clinical and biochemical parameters in polycystic ovary syndrome (PCOS) patients.

**Methods:**

In this randomized, double‐blind, placebo‐controlled clinical trial, 84 PCOS patients were randomly assigned into the treatment (90 µg Menaquinone‐7 daily for 8 weeks) and placebo groups. Insulin resistance, lipid profile, endocrine biomarkers, and body composition of the participants were measured before and after the intervention. This study was performed in Ghadir Mother & Child Hospital affiliated to Shiraz University of Medical Sciences, Shiraz, Iran.

**Results:**

Menaquinone‐7 supplementation, when compared to placebo, significantly decreased serum fasting insulin (*p* = .002), homeostasis model of assessment insulin resistance (*p* = .002), and homeostasis model of assessment β‐cell function (*p* = .02) in addition to a significant increase in quantitative insulin sensitivity check index (*p* = .001). Also, MK‐7 administration led to significant declines in serum triglyceride (*p* = .003) and dihydrotestosterone (DHT; *p* = .03) levels, free androgen index (*p* < .001), waist circumference (*p* = .03), and body fat mass (*p* < .001) as well as significant increases in skeletal muscle (*p* < .001) and sex hormone binding globulin (SHBG, *p* < .001).

**Conclusions:**

This study highlights the beneficial effects of MK‐7 on insulin resistance, fat mass, skeletal muscle, and serum levels of triglyceride, DHT, and SHBG in PCOS patients. Therefore, it seems that MK‐7 supplementation might be an appropriate additive treatment for PCOS patients.

## Graphical abstract

The aim of this randomized, double‐blind, placebo‐controlled clinical trial was to investigate the effect of oral vitamin K2 (Menaquinone‐7) on some clinical and biochemical parameters in Polycystic OvarySyndrome (PCOS) patients. Results showed the beneficial effect of 8‐week supplementation with Menaquinone‐7 on insulin resistance, fat mass, skeletal muscle, and serum levels of triglyceride, DHT, and SHBG in the study participants.

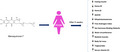

## 1 INTRODUCTION

Polycystic ovary syndrome (PCOS) is one of the most common endocrine disorders affecting women of child‐bearing age (Sirmans & Pate, 2014).

The prevalence of PCOS has been reported between 9% and 18% depending on the diagnostic criteria (Asuncion et al., 2000; Azziz et al., 2004; March et al., 2010). Moreover, 15.2% of Iranian women have been estimated to be affected with this syndrome (Mehrabian, Khani, Kelishadi, & Ghanbari, 2011). Hyperandrogenism, oligo/anovulation, and ovaries with polycystic features are the main characteristics of PCOS according to Rotterdam criteria (Jahromi, Dabaghmanesh, Parsanezhad, & Fatehpoor, 2017).

Higher rates of insulin resistance, dyslipidemia, obesity, endothelial dysfunction, and systemic inflammation, which might increase the risks of type 2 diabetes and cardiovascular diseases, in addition to reproductive dysfunction have been reported in PCOS patients (Sirmans & Pate, 2014). Although the underlying etiology of PCOS is not clearly understood, insulin resistance has been suggested as the major cause (Diamanti‐Kandarakis & Dunaif, 2012).

Insulin resistance (IR) in PCOS patients results in hyperinsulinemia which increases luteinizing hormone (LH) secretion from the pituitary gland as well as androgen production from theca cells. In addition, this hyperinsulinemia decreases the hepatic generation of sex hormone binding globulin (SHBG) (Sirmans & Pate, 2014).

The primary therapeutic strategy for managing PCOS depends on the specific complaint; however, the treatment of PCOS is multidirectional. In order to improve IR, some studies advocate weight reduction and others suggest insulin sensitizers and/or antiandrogen agents (King, 2006).

There is a growing interest in the potential health benefits of micronutrients, such as vitamin K, for managing IR‐related diseases (Schwalfenberg, 2017). Several studies have suggested that vitamin K can positively affect glucose metabolism by improving insulin sensitivity and β‐cell function through increasing adiponectin expression and β‐cell proliferation, respectively (Lee et al., 2007). Karamali et al. (2017) and Razavi et al. (2016) showed that vitamin D, K, and calcium cosupplementation could improve insulin metabolism and decrease serum levels of dehydroepiandrosterone sulfate (DHEAS) and free testosterone in PCOS patients. However, Pal et al. (2012) reported the decreased serum levels of total testosterone and androstenedione after vitamin D and calcium cosupplementation in PCOS patients. To our knowledge, no study has evaluated the effect of vitamin K alone on PCOS patients. In the current study, we investigated the effect of oral vitamin K (K2, Menaquinone‐7) supplementation on clinical manifestations, endocrine and glycemic indices, lipid profile, and body composition in PCOS patients.

## 2 MATERIALS AND METHODS

### 2.1 Study population

In the present study, all of the participants were PCOS patients recruited from the Infertility Clinic of Ghadir Mother & Child Hospital affiliated to Shiraz University of Medical Sciences, Shiraz, Iran, from July to September 2016.

Eligible participants were 18–40 years old PCOS women diagnosed based on the Rotterdam criteria (Fr & Tarlatzis, 2004). Pregnant and lactating women as well as patients with thyroid disorders, congenital adrenal hyperplasia, Cushing syndrome, diabetes mellitus, and hypertension were not involved in this study. Patients who had followed a specific diet or physical activity plans during three months prior to onset of the study were not included as well. The participants had not used any antibiotics, anticoagulants such as warfarin, supplements, or medications that were likely to affect IR, lipid profile, bone metabolism, or ovarian function in the three‐month period prior to the study.

The current study was performed based on the Declaration of Helsinki and good clinical practice guidelines. The protocol of this study was reviewed and approved by the local Ethics Committee of Shiraz University of Medical Sciences, Shiraz, Iran (IR.SUMS.REC.1396.6), and registered at the Iranian Registry of Clinical Trials (IRCT2017092836204N2).

All participants were informed about the protocol of the study, and informed consent was obtained from all individual participants included in the study. The sample size was determined to be 37 subjects in each study group according to the decrease in serum glucose level based on the results of a previous study (Rasekhi et al., 2015), with 80% power and a significance level of .05. Ultimately, 42 patients participated in each study arm to accommodate the 15% dropout rate.

### 2.2 Study design

**Figure 1:**
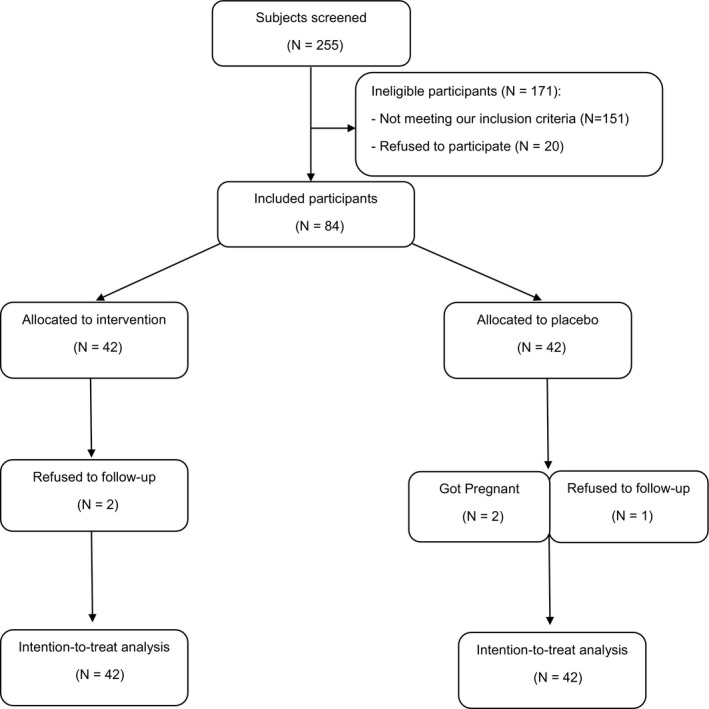
Flow diagram of patients

In this randomized, double‐blind, placebo‐controlled clinical trial, 255 PCOS patients were screened and 84 eligible participants were eventually selected which is illustrated by the Consort diagram (Figure 1).

Polycystic ovary syndrome participants were randomized into the control and treatment arms by block randomization with fixed block size of four by means of random allocation software (Saghaei, 2004). The treatment group consumed one vitamin K2 capsule, containing 90 µg of Menaquinone‐7 (MK‐7), daily for 8 weeks; the dosage was administered in a previous study (Razavi et al., 2016). Patients in the control group received a placebo capsule (avesil) daily for 8 weeks. The MK‐7 and placebo capsules were identical in color (white), smell, size (size 0), and weight which were prepared for this study by Arian Salamat Sina Company to blind the participants, researchers, and the Infertility Clinic staff to group allocation (Fermented soybean called natto was the food source of MK‐7 used in this study). A booklet containing PCOS dietary recommendation was given to both groups. The participants were requested not to change their regular physical activity level and diet and in the course of study.

To assess the participants’ adherence, a short text message was sent to their cellphone numbers on a daily basis, and they were also asked for returning their supplement package at the end of the trial. The participants were considered as adherent if they consumed 90% of MK‐7/placebo capsules. The participants were also requested to report any side effects during the study.

All the participants recorded three‐day dietary records at the beginning and in the last week of the trial. Daily energy, macronutrient (carbohydrate, protein, and fat), and micronutrient (vitamin K, vitamin D) intake of the participants were computed using Nutritionist‐4 software (First Data Bank) which was modified for Iranian food. The physical activity level was determined using the validated version of International Physical Activity Questionnaire (IPAQ) with MET‐min/week measure at baseline and at the end of the trial. Hirsutism and acne questionnaires (Banaszewska, Wrotyńska‐Barczyńska, Spaczynski, Pawelczyk, & Duleba, 2016) were also completed to measure hirsutism and acne scores of the patients before and at the end of this 8‐week intervention.

#### 2.2.1 Anthropometric assessments

At the onset of this trial and after 8 weeks of intervention, following an overnight fasting status, height (m) and weight (kg) of both groups were measured by Seca Scales to the nearest 0.1 cm and 0.1 kg, respectively. Also, based on weight and height of the participants, body mass index (BMI) was calculated [weight (kg)/height^2^ (m^2^)]. Between the lower ribs and iliac crest was determined as waist circumference and it was measured by a flexible measuring tape to the nearest 0.1 cm before and after the intervention.

Some of body composition parameters including fat mass and skeletal muscle were measured using bioelectric impedance analysis (BIA, InBody S10) in supine position at baseline and after the trial. Participants were asked not to perform any strenuous physical activity and not to be over‐ or underhydration status for at least 12 hr before their body composition analysis (Mialich, Sicchieri, & Junior, 2014).

#### 2.2.2 Blood sampling and biochemical assessments

Seven‐milliliter venous blood sample was collected after 12 hr of fasting before and after an 8‐week intervention on the third day of the follicular phase of a natural cycle from each participant. After that, for 10 min, blood samples were centrifuged at 2,000 g/min; the separated sera were frozen at −80°C until further analyses.

The primary aim of this study was to evaluate the changes in fasting serum glucose level from the beginning until the end of the trial in an intention‐to‐treat population. Serum levels of glucose and lipid profile were measured via an autoanalyzer device (Biotecnica Instrument, BT1500), using commercial diagnostic kits (Parsazmun Co). ELISA kits (Parsazmun ) were utilized to determine serum levels of follicular‐stimulating hormone (FSH) and luteinizing hormone (LH). Serum levels of insulin, sex hormone binding globulin (SHBG), total testosterone, and dehydroepiandrosterone sulfate (DHEAS) were also detected by ELISA kits (Monobind). Dihydrotestosterone (DHT) and vitamin K levels were measured by ELISA kits as well (LDN, and ZellBio, respectively). The suggested formulas were used to calculate free androgen Index (FAI), quantitative insulin sensitivity check index (QUICKI), homeostasis model of assessment β‐cell function (HOMA‐β), and homeostasis model of assessment insulin resistance (HOMA‐IR) (Hosseinzadeh, Hosseinzadeh‐Attar, Yekaninejad, & Rashidi, 2016; Pisprasert, Ingram, Lopez‐Davila, Munoz, & Garvey, 2012).

### 2.3 Statistical analysis

Statistical Package for the Social Sciences, version 21 (SPSS, Inc.), was used to perform statistical analysis. According to normal/abnormal distribution, the data were reported as median (IQR) or mean ± *SD*.

Kolmogorov–Smirnov and Levene's tests were used to check normal/abnormal distribution and homoscedasticity of the data. For comparing between the groups, independent sample *t* test was used for normally distributed data, and Mann–Whitney *U* test compared skewed data. Comparing the means within each group of the study was done by a paired *t* test or Wilcoxon test. The *p*‐value of .05 or less than .05 was statistically significant.

## 3 RESULTS

Eighty‐four PCOS patients who met the inclusion criteria participated in this clinical trial. Two patients in the vitamin K group were withdrawn from the study due to lack of follow‐up, and three patients in the placebo group did not complete the study; one patient due to lack of follow‐up and the other two became pregnant (Figure 1).

The patients’ compliance rate was more than 90% in both groups of the study. No adverse effect was reported in the course of study.

Table 1 reports baseline characteristics of the patients; no significant differences can be seen between the study arms in terms of anthropometric measurements, age, biochemical parameters, and acne and hirsutism scores.

**Table 1:** Baseline characteristics in both groups of the study (data are presented as mean ± *SD* or median [IQR])

Variable	Menaquinone‐7 (*n* = 42)	Placebo (*n* = 42)	Comparison between groups (*p*‐Value)^a^
Age (year)	28 (26–30)	27 (24–28)	.71
Weight (kg)	68.02 ± 11.28	70.47 ± 14.08	.39
BMI(kg/m^2^)	25.93 ± 4.18	27.31 ± 4.78	.16
Waist circumference (cm)	87 (82.65–100.17)	90 (84.25–98.9)	.64
Fat mass (kg)	24.08 ± 7.22	25.83 ± 9.08	.34
Skeletal muscle (kg)	24.22 ± 3.8	24.54 ± 3.8	.7
Hirsutism score	6 (2–11.75)	5 (2–9)	.49
Acne score	2 (1–2)	2 (1–3)	.22
Physical activity (MET‐min/week)	594 (198–1282.5)	462 (231–997.5)	.5
LH (IU/L)	9.7 (7.52–10.8)	9.7 (9–10.8)	.77
FSH (IU/L)	4.75 (4.4–5)	5 (4.5–5.7)	.09
Total testosterone (ng/ml)	0.84 (0.75–0.9)	0.8 (0.72–0.89)	.58
Fasting glucose (mg/dl)	90.32 ± 7.52	89.19 ± 8.5	.52
Fasting insulin (µIU/ml)	8.05 (7–9)	7.2 (6.5–8.65)	.19
Serum vitamin K (ng/ml)	436.95 (400–495.7)	434.3 (401.2–492.02)	.85

Note

*p*‐value < .05 was considered as significant level of differences.

Abbreviations: BMI, body mass index; DHEAS, dehydroepiandrosterone sulfate; DHT, dihydrotestosterone; FAI, free androgen index; FSH, follicular‐stimulating hormone; HOMA‐IR, homeostasis model of assessment insulin resistance; HOMA‐β, homeostasis model of assessment β‐cell function; LH, luteinizing hormone; QUICKI, quantitative insulin sensitivity check index; SHBG, sex hormone binding globulin.

^a^ Obtained from independent samples *t* test (for parametric data) or Mann–Whitney *U* test (for nonparametric data).

Table 2 shows anthropometric measurements and clinical findings before and after 8 weeks of the intervention. According to this table, waist circumference (*p* = .03) and fat mass (*p* < .001) decreased significantly in vitamin K group compared to the placebo group, and skeletal muscle mass (*p* < .001) increased significantly in the intervention group compared to the controls. However, there were not any significant differences in weight, BMI, hirsutism and acne scores, and physical activity level between the groups after the eight‐week intervention (Table 2).

**Table 2:** Comparison of anthropometric and clinical parameters of the participants at baseline and after 8 weeks of intervention (data are presented as mean ± *SD* or median [IQR])

Variable	Menaquinone‐7 (*n* = 42)			Placebo (*n* = 42)			*p*‐Value^b^
	Baseline	8 weeks	*p*‐Value^a^	Baseline	8 weeks	*p*‐Value^a^	
Weight (kg)	68.02 ± 11.28	67.87 ± 11.48	.82	70.47 ± 14.08	70.07 ± 14.08	.7	.97
BMI (kg/m^2^)	25.93 ± 4.18	25.91 ± 4.25	.71	27.31 ± 4.78	27.22 ± 4.86	.45	.76
Waist circumference (cm)	87 (82.65–100.17)	86.25 (82.27–98.8)	.08	90 (84.25–98.9)	90 (84.2–98.8)	.007	.03
Fat mass (kg)	24.08 ± 7.22	22.97 ± 7.34	<.001	25.83 ± 9.08	26.2 ± 9.05	<.001	<.001
Skeletal muscle (kg)	24.22 ± 3.8	24.6 ± 3.7	.003	24.54 ± 3.8	24.17 ± 3.74	.049	<.001
Hirsutism score	6 (2–11.75)	6 (2–11.75)	1	5 (2–9)	5 (3–10)	1	1
Acne score	2 (1–2)	2 (1–2)	.31	2 (1–3)	2 (1–3)	.56	.26
Physical activity (MET‐min/week)	594 (198–1282.5)	649 (347–1282.5)	.5	462 ( 231–997.5)	594 (231–1,688)	.02	.38

Note

*p*‐value < .05 was considered as significant level of differences.

Abbreviation: BMI, body mass index.

^a^ Obtained from pair *t* test (for parametric data) or Wilcoxon (for nonparametric data).

^b^ Obtained from independent samples *t* test (for parametric data) or Mann–Whitney (for nonparametric data).

In comparison with the placebo, 8 weeks of supplementation with vitamin K improved serum vitamin K level (*p* < .001) (Table 3). This study also showed that vitamin K supplementation significantly reduced TG (*p* = .003), fasting insulin (*p* = .002), HOMA‐IR (*p* = .002), HOMA‐β (*p* = .02), and increased QUICKI index compared to placebo (0.003 vs. 0.002, *p* = .001). In addition, the increased level of fasting serum glucose was higher than the control group compared with the vitamin K group (*p* = .05 vs. *p* = .92 ; Table 3).

**Table 3:** Endocrine parameters, lipid profile, and glycemic indices at baseline and after 8 weeks of intervention (data are presented as mean ± *SD* or median [IQR])

Variable	Menaquinone‐7 (*n* = 42)			Placebo (*n* = 42)			*p*‐Value^b^
	Baseline	8 weeks	*p*‐Value^a^	Baseline	8 weeks	*p*‐Value^a^	
LH (IU/L)	9.7 (7.52–10.8)	9.8 (7.5–10.65)	.66	9.7 (9–10.8)	9.65 (8.45–10.82)	.07	.11
FSH (IU/L)	4.75 (4.4–5)	4.75 (4.4–5)	.31	5 (4.5–5.7)	4.9 (4.5–5.92)	.88	.94
Total testosterone (ng/ml)	0.84 (0.75–0.9)	0.88 (0.76–0.91)	.13	0.8 (0.72–0.89)	0.86 (0.75–0.9)	.12	.95
SHBG (nmol/L)	50.2 (43.15–70.25)	55.1 (45.35–73.35)	<.001	50.9 (34.55–70.6)	50 (33.2–77)	.6	<.001
FAI	0.05 (0.04–0.06)	0.04 (0.03–0.05)	<.001	0.05 (0.03–0.08)	0.05 (0.03–0.09)	<.001	<.001
DHEAS (µg/ml)	3.42 ± 0.83	3.33 ± 0.86	.15	3.14 (2.69–3.76)	3 (2.53–3.9)	.75	.48
DHT (pg/ml)	266.9 (216.35–287)	253.7 (209.27–280.3)	.12	260.6 (222.5–288.2)	267.5 (227.5–292.05)	.05	.03
Total cholesterol (mg/dl)	165.9 ± 30.98	174.67 ± 38.7	.001	171.07 ± 31.43	172.97 ± 38.13	.09	.1
LDL cholesterol (mg/dl)	91 (83.5–107.75)	91.5 (85–107.5)	.56	96 (78–109.5)	93 (77–110.25)	.49	.34
HDL cholesterol (mg/dl)	49 (43–56.75)	49.5 (42.25–54)	.05	49 (40.5–54)	47.5 (41–54)	.78	.1
Triglycerides (mg/dl)	128.55 ± 49.37	125.52 ± 48.46	.04	122.12 ± 37.09	124.84 ± 38.3	.16	.003
Fasting glucose (mg/dl)	90.32 ± 7.52	90.35 ± 7.39	.92	89.19 ± 8.5	90.18 ± 8.05	.05	.21
Fasting insulin (µIU/ml)	8.05 (7–9)	7.4 (6.4–8.6)	.001	7.2 (6.5–8.65)	8 (6.9–8.9)	.24	.002
HOMA‐IR	1.71 (1.55–2.03)	1.47 (1.23–1.75)	<.001	1.6 (1.46–1.8)	1.56 (1.35–1.66)	.001	.002
HOMA‐β	6 (4.8–6.83)	5.59 (4.61–6.59)	.003	5.68 (4.85–7.88)	6.11 (4.87–7.75)	.71	.02
QUICKI	0.35 (0.34–0.35)	0.35 (0.34–0.36)	.002	0.35 (0.34–0.36)	0.35 (0.34–0.35)	.12	.001
Serum vitamin K (ng/ml)	436.95 (400–495.7)	450.2 (429.4–511.4)	<.001	434.3 (401.2–492.02)	420.25 (393.87–480)	.01	<.001

Note

*p*‐value < .05 was considered as significant level of differences.

Abbreviations: DHEAS, dehydroepiandrosterone sulfate; DHT, dihydrotestosterone; FAI, free androgen index; FSH, follicular‐stimulating hormone; HOMA‐IR, homeostasis model of assessment insulin resistance; HOMA‐β, homeostasis model of assessment β‐cell function; LH, luteinizing hormone; QUICKI, quantitative insulin sensitivity check index; SHBG, sex hormone binding globulin.

^a^ Obtained from pair *t* test (for parametric data) or Wilcoxon (for nonparametric data).

^b^ Obtained from independent *t* test (for parametric data) or Mann–Whitney (for nonparametric data).

Table 3 indicates that although vitamin K supplementation in PCOS patients led to significant reductions in FAI and serum DHT level (*p* < .001 and *p* = .03, respectively), it significantly increased serum SHBG concentration compared to the control arm (*p* < .001).

Having said all that, we did not observe any significant effect of vitamin K supplementation on fasting serum glucose, endocrine parameters, and other components of serum lipid profile.

In addition, there was no significant difference between the groups at baseline and end‐of‐trial dietary macro‐ and micronutrient intake (Energy, protein, fat, carbohydrate, fiber, vitamin K, and vitamin D).

## 4 DISCUSSION

The current study is the first to assess the effect of vitamin K supplementation on PCOS clinically. The results showed the beneficial effects of 8 weeks of MK‐7 (a derivative of vitamin K with high bioaccessibility and long half‐life in the form of K2) supplementation on DHT hormone, FAI, SHBG, serum fasting insulin, HOMA‐IR, HOMA‐B, QUICKI, and some anthropometric parameters including waist circumference, fat mass, and skeletal muscle in PCOS patients.

Insulin resistance, as a key factor in the development of PCOS, can result in secondary metabolic disorders such as type 2 diabetes and endothelial dysfunction (Sirmans & Pate, 2014). In the present study, after 8 weeks of supplementation with 90 µg/day of MK‐7, significant reductions in fasting serum insulin, IR, and pancreatic β‐cell function were observed, while QUICKI (as an insulin sensitivity index) increased significantly. Moreover, the increase in fasting serum glucose level was greater in the control group compared to the intervention arm. These findings indicated that vitamin K (MK‐7) consumption increased insulin sensitivity, decreased the pressure on pancreatic β‐cells, and significantly decreased insulin secretion. In line with these results, Asemi et al. (2016) and Karamali et al. (2017) reported significant decreases in insulin concentrations, HOMA‐IR, and HOMA‐β and a significant increase in QUICKI with vitamin D, K, and calcium cosupplementation after 8 and 12 weeks of the intervention in PCOS and diabetic patients, respectively. Furthermore, Rasekhi et al. (2015) observed improvements in insulin sensitivity index and glycemic status in prediabetic nonpostmenopausal women after 4 weeks of vitamin K (K1, Phylloquinone) supplementation. Also, consumption of 30 mg menatetrenone (vitamin K2) per day by young healthy men for 4 weeks caused optimal alterations in insulin sensitivity index (Choi et al., 2011). Moreover, a direct correlation has reported between vitamin K (phylloquinone) intake and appropriate glycemic status in both men and women (Yoshida, Booth, Meigs, Saltzman, & Jacques, 2008). In contrast to these studies, Kumar, Binkley, and Vella (2010) reported that daily consumption of 1 mg phylloquinone (vitamin K1) by healthy postmenopausal women caused no significant changes in glucose metabolism. In addition, Yoshida, et al. (2008) demonstrated that daily supplementation with 500 mg phylloquinone for 36 months in menopausal women did not result in any significant difference on glycemic status. The contradictory results of these studies might be due to the decrease in insulin sensitivity along with aging in postmenopausal women (Krentz, 2008).

The exact molecular mechanism of how vitamin K affects human glycemic status is not completely understood; however, some preclinical studies have shown the important role of osteocalcin (bone Gla protein) in this regulation (Ferron, Hinoi, Karsenty, & Ducy, 2008; Lee et al., 2007). Vitamin K is a fundamental cofactor for the carboxylation of osteocalcin (Rochefort et al., 2011).

Osteocalcin promotes β‐cell proliferation and insulin secretion and contributes to regulation of glycemic status as a mediator between the bones and pancreas. Moreover, osteocalcin helps to increase insulin sensitivity through adiponectin expression in fat cells (Lee et al., 2007). Lee et al. (2007) showed that the lack of osteocalcin gene resulted in some metabolic dysfunction such as hyperglycemia, glucose intolerance, increasing fat mass and serum triglyceride level, and decreasing β‐cell production, insulin secretion, and adiponectin expression.

There is a controversy about the form of osteocalcin (carboxylated or undercarboxylated) which affects the glycemic status. According to several animal studies, undercarboxylated osteocalcin (ucOC) plays a role in regulating glucose metabolism (Ferron et al., 2008; Lee et al., 2007); however, some human studies reported that serum levels of total osteocalcin or carboxylated osteocalcin (cOC) were responsible for improving glycemic status and more appropriate body composition (Knapen et al., 2012; Kunutsor, Apekey, & Laukkanen, 2015). This inconsistency may partly be due to the differences between animals and human in terms of osteocalcin gene expression and carboxylation mechanisms (Manna & Kalita, 2016).

Fat tissue abnormalities (such as defective glucose transfer and adiponectin gene expression) in addition to an increase in body fat have been reported to be associated with defective insulin activity in PCOS patients (Chazenbalk et al., 2010; Chen et al., 2013; Diamanti‐Kandarakis, 2007). On the other hand, serum vitamin K level was reported to be lower in women with a higher body fat mass (Shea et al., 2010). The present study showed that 8 weeks of MK‐7 supplementation significantly reduced waist circumference and body fat mass along with increasing skeletal muscle. In line with these findings, in an In vivo study, MK‐4 could affect the reduction of fat deposition through adiponectin regulation (Sogabe, Maruyama, Baba, Hosoi, & Goseki‐Sone, 2011). Moreover, an in vitro study has reported the inhibitory role of Menaquinone‐4 (MK‐4) in adipocyte formation in bone marrow cells (Takeuchi, Suzawa, Fukumoto, & Fujita, 2000). In addition, Knapen et al. (2012) showed that supplementation with MK‐4 in menopausal women for 3 years had a protective effect against increasing body weight and BMI. They also reported significant reverse correlations between serum vitamin K level and waist circumference before their intervention. However, Shea, Dawson‐Hughes, Gundberg, and Booth (2017) reported that 3 years of vitamin K supplementation in the elderly subjects had no significant positive effect on their body composition. Because of the contradictory results of these studies as well as the limited number of papers related to this subject, more studies are needed to recognize vitamin K functional mechanisms in regulating body composition.

The present study also recorded the reduced serum DHT level, increased serum SHBG level, and a reduction in FAI after MK‐7 administration. In line with these findings, Razavi et al. (2016) showed that 8 weeks of vitamin D‐K‐calcium cosupplementation in PCOS patients significantly reduced serum free testosterone concentration. A number of studies on PCOS found that the IR development in these patients could increase androgen secretion from ovarian theca cells, decrease liver secretion of SHBG, and increase FAI (Azziz et al., 2004). Therefore, it appears that the reduction in body fat mass in the intervention group could improve insulin resistance, which might lead to the decreased DHT and increased SHBG.

Metabolic abnormalities in PCOS patients such as IR, hyperandrogenism, and increased central fat can worsen each other and cause the development of dyslipidemia (Diamanti‐Kandarakis, Papavassiliou, Kandarakis, & Chrousos, 2007). In fact, dyslipidemia is common in these patients and can increase the risk of cardiovascular diseases with aging (Wild, 2012). The present study showed that 8 weeks of MK‐7 supplementation in PCOS patients caused a significant reduction in serum TG (but not other lipid profile components). Also, Karamali et al. (2017) reported a significant decrease in serum TG level in PCOS patients after consuming vitamin K‐D‐calcium for 8 weeks. Moreover, a number of rodent studies observed the positive effects of vitamin K on improving lipid metabolism (Kawashima et al., 1997; Sogabe et al., 2011). However, Knapen et al. (2015) showed that the consumption of yogurt fortified with MK‐7 by a healthy population had no significant effect on serum lipid levels. Furthermore, Kolahi, Pourghassem Gargari, Mesgari Abbasi, Asghari Jafarabadi, and Ghamarzad Shishavan (2015) reported no significant effect of vitamin K supplementation on lipid profile in women with rheumatoid arthritis. The reduced serum TG level in this study might have been due to the improvements in IR, FAI, and fat mass.

Since the basis of all abnormalities in PCOS is probably the low‐grade systemic inflammation (Adams et al., 2016), it should be considered that the potential effects of vitamin K on improving inflammatory status could also contribute to at least a part of the positive results in this study (Ohsaki et al., 2010; Reddi et al., 1995; Shea et al., 2007). Unfortunately, due to limited funding, lack of measuring inflammatory markers and adiponectin (as the factors involved in vitamin K molecular mechanisms in the human body) are the limitations of the present study. In addition, it would have been more appropriate to determine serum levels of osteocalcin isoforms (carboxylated and undercarboxylated forms) before and after the intervention to better interpret the alterations. Measuring these markers is recommended along with long‐term interventions in future studies. Moreover, in vitro and in vivo studies seem to be necessary for figuring out the role of osteocalcin signaling pathways in regulating sex steroid production in PCOS.

## 5 CONCLUSION

This study showed the beneficial effects of vitamin K (MK‐7) on the reduction of insulin resistance, body fat mass, and triglycerides, and increasing skeletal muscle mass, serum DHT, and SHBG levels in patients with PCOS. It appears that such supplementation might be an appropriate additive treatment for PCOS. Therefore, more studies with longer periods of intervention are needed to confirm the beneficial effects of vitamin K in these patients.

## CONFLICT OF INTEREST

The authors declare they do not have any conflict of interest.

## ETHICAL APPROVAL

The current study was performed based on the Declaration of Helsinki and good clinical practice guidelines. This study was approved by the local Ethics Committee of Shiraz University of Medical Sciences, Shiraz, Iran (IR.SUMS.REC.1396.6). Written informed consent was received and documented for all study participants.

## Data Availability

The data that support the findings of this trial are available from the corresponding author, N.H, upon reasonable request.
